# Trend of antibiotics usage for acute pyelonephritis in Korea based on national health insurance data 2010–2014

**DOI:** 10.1186/s12879-019-4191-0

**Published:** 2019-06-25

**Authors:** Bongyoung Kim, Rangmi Myung, Myoung-jae Lee, Jieun Kim, Hyunjoo Pai

**Affiliations:** 1 Department of Internal Medicine, College of Medicine, Hanyang University, 222 Wangsimni-ro, Seongdong-gu, Seoul, 04763 Korea; 2 Department of Economics, College of Political Science & Economics, Korea University, 145 Anam-ro, Seongbuk-gu, Seoul, 02841 Korea

**Keywords:** Acute pyelonephritis, Antibiotic consumption, Resistance, Stewardship, National health insurance, Korea

## Abstract

**Background:**

The objective of this study is to describe the changes in prescribing practices of antibiotics to treat acute pyelonephritis (APN) in Korea.

**Methods:**

The claim data base of the Health Insurance Review and Assessment Service in Korea was used to select patients with ICD-10 codes N10 (acute tubulo-interstitial nephritis) or N12 (tubulo-interstitial nephritis, not specified as acute nor chronic) as the primary discharge diagnosis during 2010–2014. Consumption of each class of antibiotics was converted to Defined Daily Dose (DDD)/event.

**Results:**

Throughout the five-year period, the average antibiotic consumption were 11.3 DDD per inpatient event and 6.0 DDD per outpatient event. The annual average antibiotic consumption increased for inpatients (*P* = 0.002), but remained stable for outpatients (*P* = 0.066). The use of parenteral antibiotics increased for inpatients (*P* < 0.001), but decreased for outpatients (*P* = 0.017). As for the the antibiotic classes, 3^rd^ generation cephalosporins (3^rd^ CEPs) was the most commonly prescribed (41.4%) for inpatients, followed by fluoroquinolones (FQs) (28.5%); for outpatient, FQs (54.8%) was the most commonly prescribed, followed by 3^rd^ CEPs (13.1%). The use of 3^rd^ CEPs (*P* < 0.001), beta-lactam/beta-lactamase inhibitors (*P* = 0.007), and carbapenems (*P* < 0.001) increased substantially for the treatment of hospitalized APN patients. In particular, carbapenems use increased 3.1-fold over the 5 years.

**Conclusions:**

Prescription of broad-spectrum antibiotics increased much for the treatment of APN in Korea during 2010–2014.

## Background

Acute pyelonephritis (APN) is one of the most common community-acquired bacterial infections, which usually require antibiotic treatment [1]. The annual incidence rate of APN is 39.1 per 10,000 adults in Korea (9.6 per 10,000 adults for inpatients; 29.4 per 10,000 adults for outpatients), and eleven times more women are diagnosed with APN than men [2]. *Enterobacteriaceae* are common uropathogen, and *Escherichia coli* is the most common among them. Uropathogens from APN have become resistant to several important antibiotics such as trimethoprim/sulfamethoxazole (SXT), fluoroquinolones (FQs) and the 3^rd^ generation cephalosporins (3^rd^ CEPs) and antibiotic resistance of uropathogens resulted in unfavorable clinical responses in community-acquired APN [3, 4]. In Korea, the resistance rates of SXT, FQs and 3^rd^ CEPs among *E. coli* for community-acquired APN were 27.8, 21.3 and 9.3%, respectively, during 2010–2012 [5]. Antibiotic use and antibiotic resistance of the pathogens are closely connected and influence each other.

The aim of this study is to describe the changes in prescribing practices of antibiotics used to treat APN during 2010–2014, using the National Health Insurance claim data in Korea, which may give a clue to the overall changes in prescribing practices of antibiotics used to treat common bacterial infections.

## Methods

### Data source

The National Health Insurance System of Korea covers almost the entire population, including low-income families receiving medical aid: 98% of the population was covered in 2014 [6]. We obtained the National Health Insurance claim data through the Healthcare Big Data Hub, where the Health Insurance Review & Assessment Service provides online health insurance data for fee. The data set includes information on age, gender, insurer type, clinic/hospital code, area code of clinic/hospital, care type (inpatient/outpatient), treatment start date, treatment days, admission or visit days, primary discharge diagnosis code, sub-discharge diagnosis code, medical department in charge, medical cost, and prescribed pharmaceuticals. The discharge diagnoses were coded following the *International Classification of Diseases*, Tenth Revision (ICD-10). Usually, there is a 1–2-month lag in billing for health insurance claims. We obtained the claim data for January 2010 to July 2015.

### Definitions

**Fig. 1:**
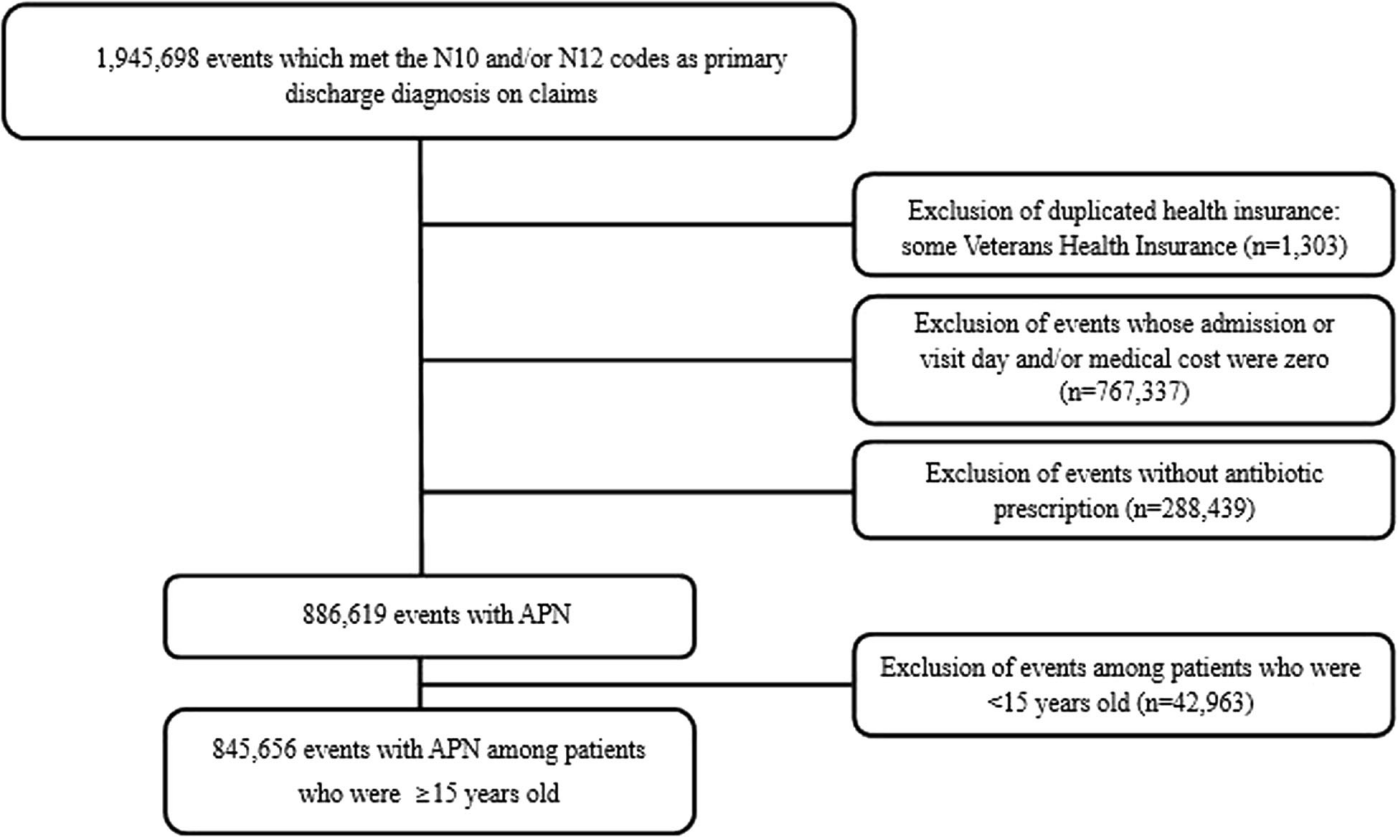
Flow diagram showing the process of selecting events with acute pyelonephritis (APN) based on the National Health Insurance claim data

We included patients whose primary discharge diagnosis was APN from 2010 to 2014: ICD-10 codes N10 (acute tubulo-interstitial nephritis) or N12 (tubulo-interstitial nephritis, not specified as acute nor chronic). Two or more claims during a 14-day period were regarded as a single event. The claims for the Veteran Health Insurance were excluded because beneficiaries of the Veteran Health Insurance could claim multiple times with a single event. The events with zero admission/visit day or zero medical cost and the events without antibiotic prescription were also excluded, as well as patients under 15 years of age (Fig. 1).

Antibiotics were defined as medication with class J01 from the Anatomical therapeutic chemical (ATC) classification system, which does not include antifungal agents nor anti-tuberculosis agents. Systemic agents with per oral or parenteral administration route were included while topical agents were excluded. The consumption of each class of antibiotics was converted to Defined Daily Dose (DDD), following ATC classification system of the World Health Organization (WHO) [7]. As for payment provider, it is either the health insurance or the medical aid; the medical aid is a national health care only for qualified low-income families. If there were more than one method of payment in record, we selected the payment provider who paid for the hospitalization prior to outpatient visit and who is involved in the earliest recorded variable.

### Data analysis and statistics

Linear regression models with monthly measured variables were used to assess the trend of antibiotic consumption over time or age. Statistical significance was defined as *P* < 0.05. All analyses were performed using SAS enterprise guide version 6.1 (SAS Institute Inc., Cary, NC).

## Results

A total of 845,656 APN events were identified during 2010–2014 in Korea (151,380 in 2010; 157,353 in 2011; 168,470 in 2012; 175,208 in 2013; 193,245 in 2014). Most cases (93.1%, 787,305/845,656) met N10 code (acute tubulo-interstitial nephritis) in ICD-10. One out of 4.1 patients were hospitalized (inpatients 208,652 and outpatients 637,004).

### Overall antibiotic consumption

**Fig. 2:**
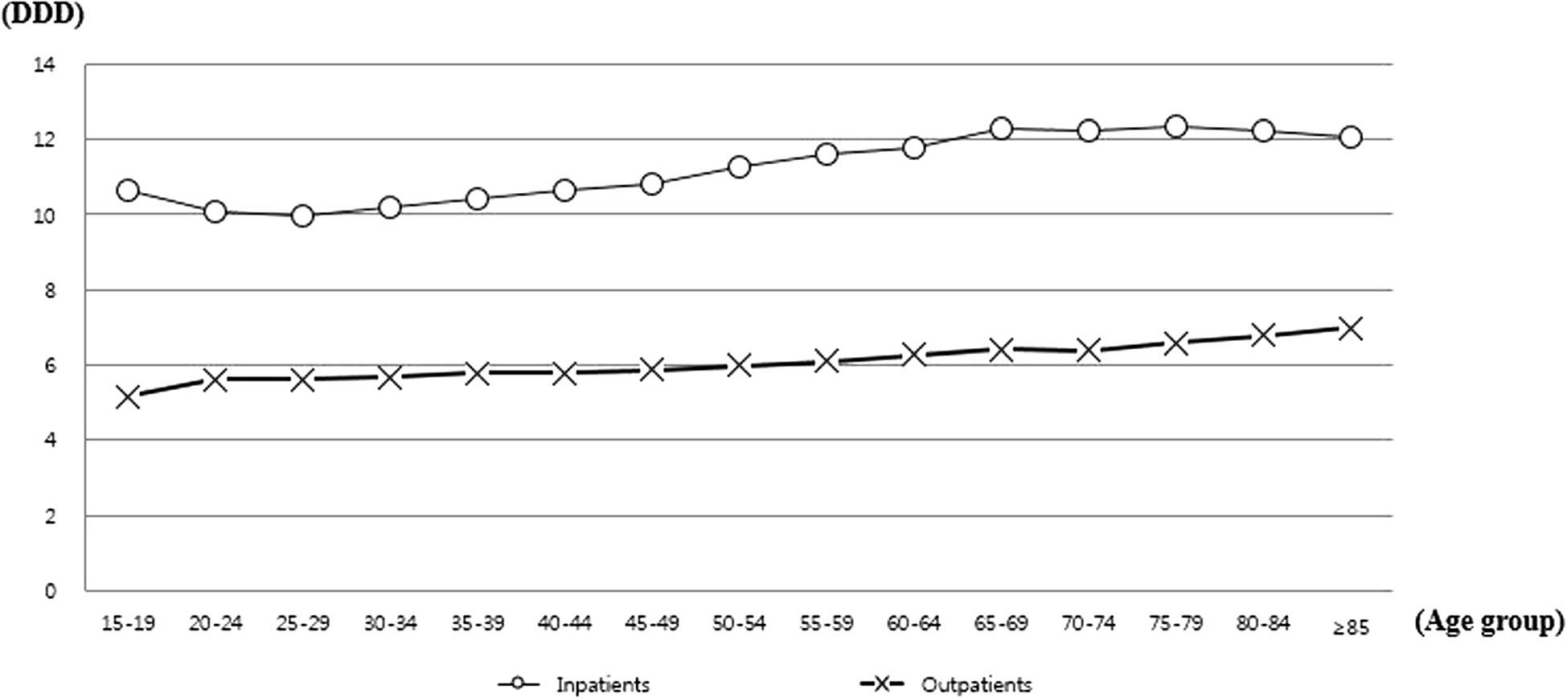
Average antibiotic consumption per acute pyelonephritis event in Korea by age group, 2010–2014

The average amount of antibiotic consumption per inpatient event was 11.3 DDD, and it increased throughout the study period (10.8 DDD in 2010, 11.1 DDD in 2011, 11.3 DDD in 2012, 11.5 DDD in 2013, 11.7 DDD in 2014, *P* = 0.002). On the other hand, the average amount of antibiotic consumption per outpatient event was 6.0 DDD, which remained stable throughout the study period (6.0 DDD in 2010, 6.1 DDD in 2011, 6.0 DDD in 2012, 6.0 DDD in 2013, 5.8 DDD in 2014, *P* = 0.066). Males received more antibiotics than females in both inpatient (males, 12.9 DDD vs. females, 10.4 DDD) and outpatient events (males, 6.3 DDD vs. females, 5.9 DDD). As for the antibiotic use by age, more antibiotics were prescribed for older patients (inpatients, *P* < 0.001; outpatients, *P* < 0.001) (Fig. 2).

Regarding payer type, beneficiaries of the medical aid consumed more antibiotics than those covered by the health insurance in inpatient events (medical aid, 12.4 DDD vs. health insurance, 11.2 DDD), but not in outpatient events (medical aid, 6.9 DDD vs. health insurance, 7.2 DDD). As for hospital type, the average amount of antibiotics usage was the highest among patients admitted to tertiary hospitals in both inpatient and outpatient events (inpatients, 13.8 DDD; outpatients, 10.0 DDD), followed by secondary hospitals (inpatients, 11.5 DDD; outpatients, 7.7 DDD).

### Antibiotic consumption by administration route

**Fig. 3:**
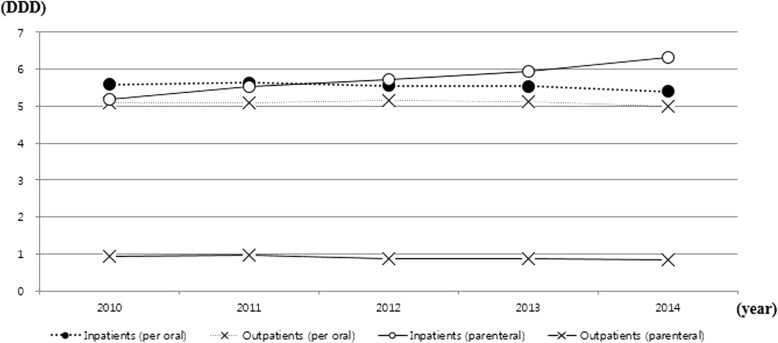
Annual average antibiotic consumption in inpatients and outpatients of acute pyelonephritis event in Korea by administration route (parenteral VS. per oral), 2010–2014

Figure 3 shows annual average antibiotic consumption per APN event by administration route. Parenteral antibiotic consumption increased throughout the study period for inpatients (5.20 DDD in 2010, 5.53 DDD in 2011, 5.71 DDD in 2012, 5.94 DDD in 2013, 6.31 DDD in 2014, *P* < 0.001), but it mostly decreased for outpatients (0.95 DDD in 2010, 0.96 DDD in 2011, 0.88 in 2012, 0.86 in 2013, 0.82 in 2014, *P* = 0.017). As for per oral antibiotics, no significant change was observed in both inpatients (*P* = 0.075) and outpatients (*P* = 0.453).

### Proportion of antibiotic classes used for the treatment of APN

**Fig. 4:**
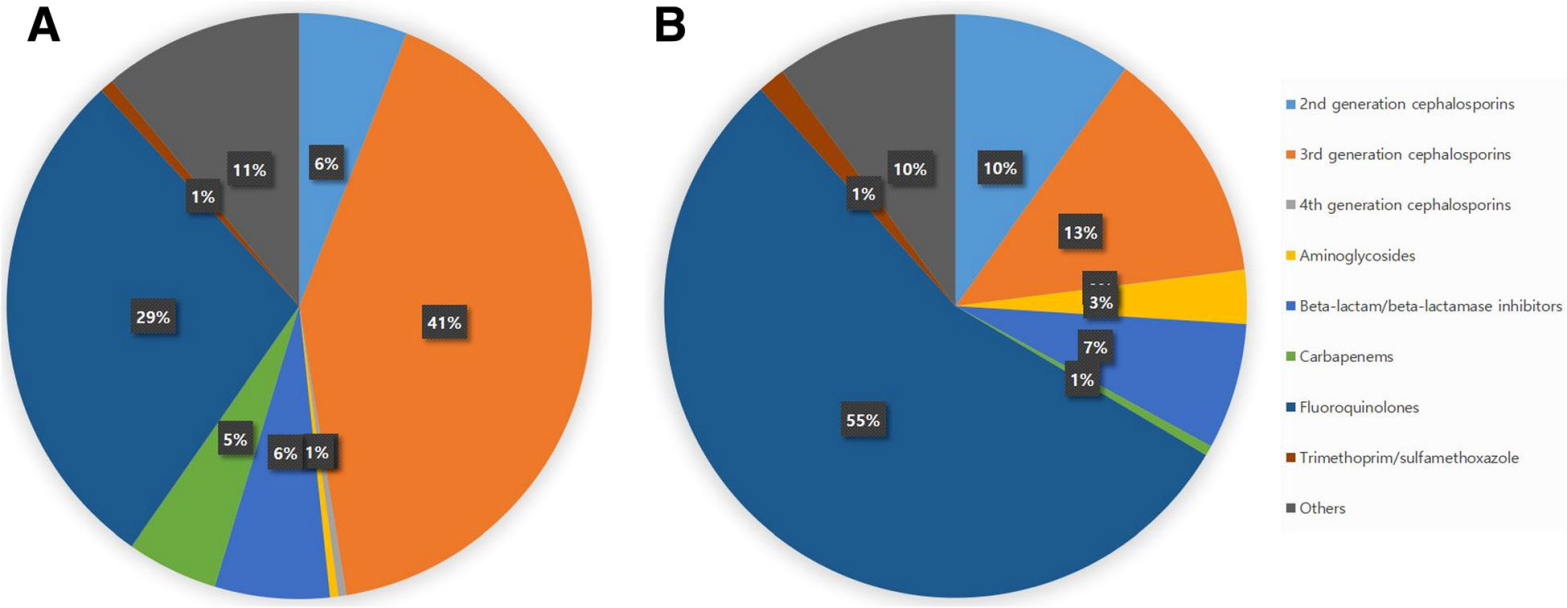
Proportion of antibiotic classes used for the treatment of acute pyelonephritis events in Korea, 2010–2014. **a** Inpatients **b** Outpatients

Figure 4 shows the proportion of antibiotic classes for the treatment of APN events from 2010 to 2014. For inpatients, 3^rd^ CEPs (4.7 DDD, 41.4%) was the most commonly prescribed, followed by FQs (3.2 DDD, 28.5%) and beta-lactam/beta-lactamase inhibitors (BL/BLIs) (0.7 DDD, 6.4%). For outpatients, FQs (3.3 DDD, 54.8%) was the most commonly prescribed antibiotics, followed by 3^rd^ CEPs (0.8 DDD, 13.1%) and 2^nd^ generation cephalosporins (2^nd^ CEPs) (0.6 DDD, 9.9%).

Table 1 presents the annual average consumption of antibiotics classes per APN event. For inpatients, the consumption amounts of 3^rd^ CEPs (*P* < 0.001), BL/BLIs (*P* = 0.007), and carbapenems (*P* < 0.001) increased every year over the study period; particularly, carbapenems use increased 3.1-fold, and 3^rd^ CEPs use increased by about 25%. In contrast, the use of FQs (*P* = 0.010), 1^st^ generation cephalosporins (1^st^ CEPs) (*P* < 0.001), aminoglycosides (AGs) (*P* = 0.004), and penicillins (*P* = 0.014) declined every year. There was no noticeable change in antibiotic usage among the remaining classes including 2^nd^ CEPs (*P* = 0.067), 4^th^ generation cephalosporins (4^th^ CEPs) (*P* = 0.162), and SXT (*P* = 0.575).

**Table 1:** Annual average consumption of antibiotics classes per acute pyelonephritis event in Korea, 2010–2014

Antibiotic class	Inpatients (DDD/event)							Outpatients (DDD/event)						
	2010	2011	2012	2013	2014	2014/2010 (%)	*P*	2010	2011	2012	2013	2014	2014/2010 (%)	*P*
1^st^ CEPs	0.64	0.57	0.51	0.44	0.36	56.3	< 0.001	0.22	0.21	0.20	0.17	0.16	72.7	0.002
2^nd^ CEPs	0.80	0.72	0.63	0.30	0.64	–	0.068	0.61	0.61	0.62	0.62	0.51	–	0.233
3^rd^ CEPs	4.11	4.45	4.68	4.94	5.14	125.1	< 0.001	0.67	0.75	0.83	0.82	0.83	123.9	0.049
4^th^ CEPs	0.05	0.05	0.05	0.05	0.06	–	0.162	–	–	–	–	–	–	–
AGs	0.07	0.06	0.06	0.04	0.03	42.9	0.004	0.24	0.22	0.17	0.15	0.13	54.2	0.002
BL/BLIs	0.67	0.71	0.71	0.73	0.75	111.9	0.008	0.43	0.42	0.42	0.43	0.39	–	0.294
Carbapenems	0.28	0.42	0.54	0.69	0.87	310.7	< 0.001	0.02	0.03	0.03	0.04	0.04	200.0	0.001
FQs	3.35	3.33	3.22	3.18	3.02	90.1	0.010	3.29	3.29	3.25	3.28	3.28	–	0.555
Penicillins	0.14	0.11	0.10	0.08	0.09	64.3	0.014	0.14	0.11	0.10	0.08	0.06	42.9	0.001
SXT	0.10	0.08	0.08	0.08	0.09	–	0.575	0.10	0.09	0.09	0.09	0.09	–	0.538

*Abbreviations*: *DDD* Defined daily dose, *1*^*st*^ *CEPs* 1^st^ generation cephalosporins, *2*^*nd*^ *CEPs* 2^nd^ generation cephalosporins, *3*^*rd*^ *CEPs* 3^rd^ generation cephalosporins, *4*^*th*^ *CEPs* 4^th^ generation cephalosporins, *AGs* Aminoglycosides, *BL/BLIs* Beta-lactam/beta-lactamase inhibitors, *FQs* Fluoroquinolones, *SXT* Trimethoprim/sulfamethoxazole

For outpatients, the use of 3^rd^ CEPs (*P* = 0.049) and carbapenems (*P* = 0.001) increased gradually (Table 1). In contrast, there was a decrease in the use of 1^st^ CEPs (*P* = 0.002), AGs (*P* = 0.002), and penicillins (*P* = 0.001). There was no noticeable change in antibiotics usage among the other antibiotics: 2^nd^ CEPs, *P* = 0.124; BL/BLIs, *P* = 0.294; FQs, *P* = 0.555; SXT, *P* = 0.538.

## Discussion

APN is one of the most common infectious diseases caused by *Enterobacteriaceae*. Because the causative organisms of APN are easy to recognize, physicians can readily respond to antimicrobial resistance when they prescribe antibiotics in treating APN. Given that the local guideline for empirical therapy of urinary tract infections (UTI) did not change during the study period, APN is an appropriate disease to examine changes in antibiotic prescribing patterns under the presence of antibiotic resistance.

In the literature, two studies examined changing antibiotic prescription patterns in APN or UTI [8, 9]. In the US, the use of SXT decreased while FQs and 3^rd^ CEPs increased among APN patients from 1997 to 2001 [8]. However, no change was seen in the annual consumption of antibiotic classes for the treatment of UTI in Italy from 2006 to 2013 [9]. In comparison to these studies, our study found that the amount of total antibiotics and parenteral antibiotics consumption per inpatient event increased over 2010–2014.

In Korea, broad-spectrum antibiotics such as 3^rd^ CEPs, BL/BLIs, and carbapenems were more prescribed in treating APN during the 5-year period than the traditional antibiotics. This probably happened for the following reasons. Firstly, the physicians were concerned about increasing multidrug-resistant pathogens in UTI. Secondly, resistance to traditional antibiotics in treating APN increased enough. Thirdly, broad-spectrum antibiotics occur more easily to the physicians’ mind.

FQs have been widely accepted as a first-line empirical antibiotic for APN in many countries [10, 11]. However, increasing resistance to FQs in urinary isolates has been reported worldwide [12–14], which might have discouraged physicians from prescribing the antibiotic as easily as they used to. Consistent with the situation of other countries, the FQs resistance rate of *E. coli* isolated from blood in large Korean hospitals was 23.9% in 2006–2007 and then increased to 30.8% in 2011 [15]. Similarly, the resistance rate of *E. coli* to FQs had increased from 21.3% in 2010–2011 to 33.5% in 2017–2018 among community-acquired APN patients [5, 16]. In discussing *E. coli* isolated from female uncomplicated cystitis, the study also demonstrated an increase in FQs resistance [17]. These changes seem to have affected the antibiotic prescription pattern for APN treatment.

We found that carbapenems use increased by 3.1-fold among inpatients (from 0.28 to 0.87 DDD/event) and by 2.1-fold among outpatients (from 0.02 to 0.04 DDD/event), respectively. Also, the proportion of carbapenems use relative to FQs (8.3% in 2010; 12.6% in 2011; 16.8% in 2012; 21.7% in 2013; 28.8% in 2014) and that to 3^rd^ CEPs (6.8% in 2010; 9.4% in 2011; 11.5% in 2012; 14.0% in 2013; 16.9% in 2014) gradually increased. Carbapenems are generally used in hospital-acquired infections caused by multi-drug resistant organisms, however, our data implicates that those broad-spectrum antibiotics are more and more commonly used in mostly community-onset relatively simple infection.

Considering the high prevalence of APN and ecological impact of the extensive use of carbapenems, this is a worrisome finding. Nationwide education and prescription control of broad-spectrum antibiotics for the treatment of common but not serious infections such as community-acquired APN should be reinforced. Other antibiotics which would replace carbapenems for the treatment of APN caused by extended-spectrum beta-lactamase (ESBL) producing organisms such as piperacillin/tazobactam or gentamicin should be studied more and recommended for the treatment of less severe APN patients [18, 19].

Broad-spectrum antibiotic consumption is a common problem in many countries. Based on the data from the European Surveillance of Antibiotic Consumption (ESAC) projects, broad-spectrum penicillins and BL/BLIs were used more frequently than traditional penicillins throughout the EU countries [20]. Carbapenems and polymyxin usage also increased substantially during 2009–2013 [20]. Likewise, the consumption of broad-spectrum antibiotics including 3^rd^ CEPs and carbapenems increased in Korea since the last decade [21]. To break out of such a vicious cycle, the establishment of antimicrobial stewardship programs should be emphasized. In China, a rapid and sustained reduction in antibiotic usage was achieved by a national antimicrobial stewardship campaign [22]. In France and Belgium, successful national antimicrobial stewardship campaigns reduced inappropriate antibiotic use for both inpatients and outpatients [23, 24]. Fortunately, the Korean Ministry of Health and Welfare launched a national action plan on antimicrobial resistance in 2016 [25]. In addition to such policies, individual hospitals should implemente antimicrobial stewardship programs which reflect each hospital’s own environment.

The main strength of the present study is documenting the increasing trend of broad-spectrum antibiotics use in treatment of APN which is a relatively simple community-acquired infection with identified pathogens in most cases. In addition, the present study is based on high-quality data source: the National Health Insurance data, which is the most representative health data in Korea. Using a large number of samples, our results show the overall changes in prescribing practices of antibiotics in a common bacterial infection clearly. Even though the reliability of diagnosis may be somewhat questionable as in other studies using administrative data, still there is no doubt that the data quality is high. APN has homogeneous pathology and simple diagnostic criteria to ease analyzing a large data set based on the entire population [26]. We also note that approximately 80% of patients whose discharge diagnosis was APN met the admission criteria in Canada [27].

Despite the above strength of this study in terms of data quality, there are also some limitations in this study. Firstly, administrative data do not contain laboratory-level microbiological data, and with the administrative data alone, we could not correlate the level of antimicrobial resistant pathogens with the increasing use of broad-spectrum antibiotic consumption. Also, due to lack of microbiological data, we could not differentiate complicated versus uncomplicated APN cases. Secondly, only the main discharge diagnosis of APN was included. Some cases might have been coded as sub-discharge diagnosis under other ICD-codes such as Gram-negative sepsis and adult respiratory distress syndrome. Thirdly, detailed information on the total duration and amount of each antibiotic use was not available, which made it impossible to analyze combined antibiotic therapies and calculate precise treatment duration for each event that are essential to judge the appropriateness of antibiotic therapies. Fourthly, using Korean data alone is certainly insufficient to shed light on the global antibiotic resistance trend in APN. Despite this limitation, learning about Korean experiences on antibiotic usage might be informative for other countries.

## Conclusions

During the 5 years from 2010 to 2014, substantially more broad-spectrum antibiotics such as 3^rd^ CEPs, BL/BLIs and carbapenems were prescribed than traditional antibiotics for the treatment of APN in Korea.

## Data Availability

The data used in this study are available from Healthcare Big Data Hub, where the Health Insurance Review & Assessment Service provides online health insurance data for fee, but restrictions apply to the availability of these data, which were used under license for the current study, and so are not publicly available. Data are available from the authors upon reasonable request if permission from Health Insurance Review & Assessment Service is obtained prior to the request.

## Abbreviations

1^st^ CEPs: 1^st^ generation cephalosporins
2^nd^ CEPs: 2^nd^ generation cephalosporins
3^rd^ CEPs: 3^rd^ generation cephalosporins
4^th^ CEPs: 4^th^ generation cephalosporins
AGs: Aminoglycosides
APN: Acute pyelonephritis
ATC: Anatomic therapeutic chemical
BL/BLIs: Beta-lactam/beta-lactamase inhibitors
DDD: Defined daily dose
ESAC: European Surveillance of Antibiotic Consumption
ESBL: Extended-spectrum beta-lactamase
ESMID: European Society for Microbiology and Infectious Diseases
ICD-10: *International Classification of Diseases*, Tenth Revision
IDSA: Infectious Diseases Society of America
SXT: Trimethoprim/sulfamethoxazole
UTI: Urinary tract infections
WHO: World Health Organization
